# Introducing Audio Podcasts into a Practical Laboratory Course for Pharmacy Students as a Novel Tool for Performance Assessment

**DOI:** 10.3390/pharmacy10020040

**Published:** 2022-03-17

**Authors:** Daniel Baecker

**Affiliations:** Department of Pharmaceutical and Medicinal Chemistry, Institute of Pharmacy, University of Greifswald, Friedrich-Ludwig-Jahn-Straße 17, 17489 Greifswald, Germany; daniel.baecker@uni-greifswald.de; Tel.: +49-3834-420-4860

**Keywords:** clinical chemistry, educast, educational podcast, mobile learning, performance assessment, pharmacy education, pharmacy students, podcast, pulse oximetry, urine test strips

## Abstract

The use of digital tools can positively impact higher education for both scholars and faculty. In recent years, it has become apparent that podcasts are a suitable medium for use in teaching. They are provided almost exclusively by lecturers for students, with students passively listening to them rather than actively participating in their production. However, this could also be valuable for students. Therefore, this pilot study investigated the extent to which the creation of a podcast would be accepted by students as a method for capturing pharmacy students’ understanding of the learning content. The evaluation was performed as part of the “Clinical Chemistry” practical course, which was attended by third-year pharmacy students in groups of three. After passing the station dealing with practical clinical chemistry relevant diagnostic systems, the groups were asked to produce an educational podcast covering the essential content on the topics of urine test strips or pulse oximetry, respectively. Student attitudes toward the adoption of podcasts as a tool for performance assessment were determined with an anonymous and voluntary survey. The respondents reported that they had fun creating the podcast, which enabled them to look at the instructional content from a different perspective. Competencies such as social and communication skills and media literacy as well as self-organized and self-directed learning were also promoted. However, the students assumed that the tool is not ideally suited for dealing with extensive topics. Nonetheless, the students clearly support the continued creation of podcasts as a performance assessment tool. In addition, they suggest integrating podcasts into other courses within the pharmacy curriculum. This may also be related to the infrequent use of novel technologies, such as podcasts, in their education thus far.

## 1. Introduction

The COVID-19 pandemic has affected every aspect of our lives. In higher education, it led to the need to redesign teaching methods [1]. Due to contact restrictions, face-to-face courses were transformed into mobile learning [2]. This change was accompanied by the demand for online and virtual tools that meet the requirements of distance learning [3]. Technological progress has long since impacted a wide range of areas of life, including education [4]. There are numerous mobile tools for distance and blended learning, among which podcasts play a leading role [5]. In recent years, the popularity of podcasting as a medium in higher education has grown tremendously [4,6,7,8]. In addition, its influence is likely to increase in areas outside the education sector [9,10].

Podcasts are indeed a medium that was originally developed as a tool for entertainment or to convey information content to the users [4]. However, the use for entertainment only has expanded, so that podcasts have also been applied as a learning tool among other fields of application [5]. Traditional podcasts provide a digital method for distributing audio content [11]. The delivery is achieved via the internet, as podcasts can be downloaded to digital devices such as computer, tablet, or smartphone [7,12]. Podcasts, which are primarily used as a method in education, are referred to as “educasts” [7]. Recording a lecture is the simplest and also the most common type of educast [5]. The recording of live lectures may additionally include video [5,13]. Podcasts designed to be watched are called “vidcasts” [5] or “vodcasts” [14].

The success of podcasts as educational tools is due to several advantages. Podcasts can be used flexibly and they enable teaching and learning without temporal or physical restrictions [4,13]. In particular, the independence from spatial boundaries shows their potential as an innovative tool during social distancing caused by the pandemic. Mobile access via portable devices leads to greater accessibility and can increase both use and motivation among students [6,15]. Aside from the convenience of listening to podcasts when and where students want (mobile learning) [14], they can use it as often as they need to [12]. The ability to pace, pause, rewind, and listen to podcasts several times, especially for content that students did not understand the first time, facilitates self-directed learning that is customized to individual learning abilities [5,7,13,16,17]. Other benefits of podcasts are their intimacy, inexpensiveness, and simplicity of production as well as fast distribution [7,10,11,14].

Podcasts are a powerful tool, at least as a supplement to traditional lectures, because students have not found them to be a substitute for live lectures [13,18]. Earlier reports on lack of institutional recognition and students’ unwillingness to use them seem to be outdated [5,15]. In addition, faculty members’ willingness to use online teaching tools depends on several parameters that were validated in a previous study [19]. The recent demand for distance learning during the pandemic has improved the perception of podcasts and endorsed its use in higher education [18]. However, the effectiveness of podcasts as a new tool substantially depends on their acceptance by students [4,6].

The use of podcasts covers many areas [15]. Strikingly, there is a substantial amount of literature reporting on the increased use of podcasts in medical student education [11,13,20,21,22] and in continuing medical education [3,8,11]. Podcasts can be incorporated in different ways, such as in the use for preparatory work, as elements to be listened to during a lecture, or to stimulate further learning [17].

Podcasts are even being implemented in medical school curricula [14], while their application in pharmacy education seems to be comparatively sparse with very few exceptions (e.g., reports on video podcasts [23,24]). Interestingly, faculty in the College of Pharmacy at Rosalind Franklin University School of Medicine and Science have created a lecture series (i.e., HelixTalk) that includes bi-monthly podcasts to help students keep up with current pharmaceutical topics (available online at: https://www.rosalindfranklin.edu/academics/college-of-pharmacy/helixtalk/, accessed on 14 March 2022).

The most common use of podcasts is to provide students with recordings of completed lectures (substitutional use), followed by the supplementary use, which aims to reinforce class content. The least frequent application is the creative use, which describes the production of podcasts by students [6]. Although educational podcasts are delivered almost exclusively to students who listen to them as passive recipients [25], creative use seems far more stimulating in terms of interaction with the learning content and its processing [13]. When students are tasked with creating an educast, they must have a profound knowledge of the instructional content acquired through active engagement with the subject [6]. This particular use of podcasts leads to more participation of students in the learning process and empowers them to develop educational content, as they actively create knowledge rather than just passively receive it [4,9].

This has given rise to the idea of introducing educasts as a performance assessment tool in a practical course as part of the pharmacy curriculum. The main focus of the current pilot study was to investigate general student perceptions of such use of podcasts, since this is the primary determinant of their effectiveness as a valuable tool in higher education.

## 2. Methods

The pilot study was performed in the winter semester 2021/22 at the Institute of Pharmacy, University of Greifswald, Germany. It was conducted as part of the practical course “Clinical Chemistry”. This course is mandatory for students enrolled in the fifth semester (third year) of a four-year pharmacy degree program (eight semesters). A total of 51 students participated in the course during the winter term 2021/22. All participants were divided into 17 groups of 3 students each, and their requests regarding composition of the particular groups could be considered. The practical course was held in attendance and was in compliance with the university’s strict hygiene regulations. The content of the course included five different stations, one of which focused on clinical chemistry relevant diagnostic systems, i.e., urine test strips, blood glucose meter, pregnancy test, COVID test, and pulse oximeter.

The methodological principles of the test systems were developed with the students in joint discussions between the docent and the three group members according to the concept of “flipped classroom”. This was quite feasible because the students had already acquired some prior knowledge on the topics, i.e., in a previous clinical chemistry lecture on the subject given earlier by another docent, through independent reading of a paper on urine test strips [26], and through other proactive preparation in order to participate in the station “testing systems”.

In addition, the students also learned about the practical use of the test systems. They analyzed artificial urine samples with the test strips and monitored the concentration of glucose solutions provided with a glucose meter. Moreover, a pregnancy self-test was performed with the previously used artificial urine samples and the students determined their arterial hemoglobin oxygen saturation and heart rate with pulse oximeters. In order to save valuable resources, the implementation of COVID self-tests was not performed in the current course. However, the students were already familiar with the practical application of these tests due to the pandemic. Therefore, only the basics of antigen- and antibody-based COVID assays were discussed in class.

In the “Clinical Chemistry” practical course, each group must submit a protocol after each station they have passed through. The submission of the protocols was expected three working days after the respective course day. Such a protocol includes outlining the basics of the applied technique, documenting experimental data, as well as evaluating and interpreting them. The station dealing with the testing systems was adduced to introduce the production of a podcast as a tool for demonstrating the academic achievement. Therefore, the groups were asked to create an audio educast that summarizes the essentials of the instructional content. The group had to decide if they wanted to create a podcast on either urine test strips or pulse oximetry. In addition to the thematic focus, students were not given any specific rules for creating the podcast (e.g., no recommendation regarding the software to be used), except that it should last 3–5 min and must be turned in after three days. Students were told that the podcasts would be evaluated on content accuracy only.

Students were invited to participate in a voluntary and anonymous survey to determine their experience and attitudes toward the use of educast as a means of performance assessment. Every student was provided with a printed questionnaire (in German) at the end of participation in the station and they were advised not to fill it out until after the podcast was created. Feedback was completely voluntary, as there was neither an incentive to return the completed questionnaire nor any disadvantage if this was not carried out. The submission was anonymous, e.g., by dropping it into the lecturer’s mailbox at the institute. The survey consisted of 27 items, which could be marked according to a 5-point Likert scale (1: strongly agree, 2: agree, 3: neutral, 4: disagree, 5: strongly disagree), determining to what extent the students agreed or disagreed with statements regarding educasts. In addition, at the end of the questionnaire, there was the possibility to enter suggestions and proposals. A descriptive qualitative analysis of the responses was conducted.

## 3. Results

The results of this pilot study are based on the analysis of the podcasts submitted by the students and on the responses received in the voluntary survey.

### 3.1. Evaluation of the Podcasts

The podcasts of all 17 groups were submitted within the prescribed time of three days via email to the docent. Although it was not used as a basis for evaluation, all educasts had good acoustic quality. Three groups added musical or acoustic effects to both the intro and the closing of their podcast. Eight groups gave their podcast a specific name, such as “No drama-pharma”, “Nice to know”, “The Pharmacy”, and “Health plus”. Aside from occasional minor inaccuracies that could be due to the challenge of proper wording during the live production of a podcast, none of the podcasts had severe errors in content. Therefore, all groups passed the performance assessment the first time.

Nine podcasts focused on urine test strips, and eight on pulse oximetry. The mean length of the submitted podcasts was 4 min and 57 s and thus met the requirement of a duration of 3–5 min. The number of speakers in five podcasts was only one person, while in the remaining twelve podcasts all three group members spoke. In the latter case, the students implemented the podcast as a coherent conversation. Classic roles were often distributed among the participants in the podcast, such as moderator-led roundtable discussions with a pharmacist, urologist (especially urine test strips), emergency medical technician (especially pulse oximetry), and a concerned patient.

### 3.2. Evaluation of the Survey

Overall, about half of all students (27 out of 51) returned the questionnaire. A detailed representation of the distribution among the answer options can be seen in the Table 1. A significant majority indicated that educational podcasts had not been used extensively in their pharmacy studies thus far (Q1), although students agreed that more novel technologies should be utilized in higher education (Q2). Respondents stated they liked podcasts in general (Q3), but they had a more neutral stance on preferring vodcasts over audio-only podcasts (Q4). However, they consented to assuming that educasts could be utilized as a tool to process instructional content (Q5).

Most survey participants indicated that they found creating an educational podcast to be a more varied method than traditionally writing a protocol (Q6). Nevertheless, a rather neutral picture emerged when it came to whether the production of a podcast succeeded in concentrating on the essential learning content (Q7). Yet, students concurred that the “creative use” of the podcast helped them in reflecting and elaborating on course content (Q8), while the effect on the learning capacity seemed rather balanced (Q9).

Nearly all participants felt that their creativity was enhanced by the task of making an educast (Q10). For this purpose, most respondents stated that they favored group work (Q11) rather than individual work (Q12). This behavior is also reflected in how many group participants actively spoke in the podcast. Although group work was preferred for an educast, the students interviewed agreed that their individual preferences were given consideration when working in groups (Q13). This eventually led to the promotion of social skills (Q14).

In addition, the respondents affirmed that communication skills, media literacy, and small group self-organization were encouraged (Q15–17). They disagreed that the time it took to produce the educast was too long (Q18). A more neutral assessment emerged regarding whether creating a podcast was a helpful exercise for skills as a future pharmacist (Q19). There was denegation among the students regarding the hypothesis that educasts should only be provided by lecturers to them (Q20).

Respondents expressed the opinion that making a podcast could be maintained as a means of performance measurement (Q21). Even further, students could imagine that the creation of a podcast could be integrated into other courses in their studies so far (Q22). However, a more neutral attitude prevailed when it came to listening to podcasts in order to prepare for final exams (Q23). The willingness to listen to educasts in their free time was also rather reserved (Q24). A neutral opinion was given regarding the use of podcasts when commuting to university (Q24a). They refused to imagine listening to educasts during sports activities (Q24b) or before going to sleep (Q24d). In contrast, the students showed a willingness to use educasts when completing household chores (Q24c).

Educast duration of 3–5 min was perceived as appropriate in this case with a very focused subject, though it would likely be of further benefit from a slight extension (Q25–26). In any case, the subject of urine test strips or pulse oximetry was judged by the respondents to be a suitable topic for the creation of an educational podcast (Q27).

In addition to the questions (Table 1), which could be answered according to a Likert scale, the students also had the opportunity to make suggestions and proposals at the end of the questionnaire. In 9 of the 27 returned questionnaires, the students made use of that option. The individual comments are listed in Table 2.

One respondent (C1) provided further information about when she/he would listen to the learning podcasts, namely at home during the learning phase. It was also argued why listening to a podcast before going to bed might not be beneficial for her/him. Another aspect was related to the question about the duration of podcasts.

Another student (C2) suggested that creating a podcast might be too laborious. Nonetheless, it was proposed that podcast technology could be incorporated into other areas of education, such as a supplement to lectures.

Two students (C3–4) praised the convenience of listening to podcasts on the side. The ability of podcasts to deal with more complicated learning content, and when it came to learning factual knowledge, was viewed critically (C3–4, C6). However, podcasts seem to be a suitable tool when the content is less extensive (such as in the current case of urine test strips and pulse oximetry) and when you want to get an overview of a topic (C4, C6), but not in the case of extensive topics (C5–6). This would require more specifications regarding the creation of the educast, although the freedom granted in the current setting was actually considered positive (C5).

Three students clearly commended the use of educasts in their comments (C7–9). They suggested using it generally more often or at least to keep it. Creating an educast ensures that students focus on the content, even from a different point of view. Moreover, it was fun for the students, which ultimately leads to increased student motivation.

## 4. Discussion

The aim of the present study was to evaluate the extent to which students accept the use of podcasts as a performance assessment tool. To this end, each group of three were required to hand in a self-prepared educast on the basics of either urine test strips or pulse oximetry after attending the respective station in the “Clinical Chemistry” practical course. This was followed by the voluntary completion of a questionnaire.

The podcasts had an average duration of about 5 min. This matches the specifications set by the docent. According to a previous classification, these podcasts are considered as short (1–5 min), compared to medium-length (6–15 min) and long (>15 min) podcasts [5]. It is generally believed that podcasts exceeding 10 min in duration are no longer useful because student attention wanes after that time [21]. On the other hand, there are reports that science-related podcasts are often rather longer than suggested [10]. This is probably due to the fact that explanations are necessary to properly deliver and grasp the content.

In about 70% of the podcasts (12 out of 17), each of the three members of the group spoke and shared in the conversation. This is consistent with the fact that students preferred to work in groups on this task. In addition, the respondents indicated that consideration of their individual ideas was possible during the elaboration, which indicates harmony and teamwork in the groups and creates a stimulating learning atmosphere. At this point, however, it should be considered that individual wishes regarding the composition of the groups were considered when students were divided into the groups. Harmony can have a positive effect on performance within a team [27]. In fact, the possibility to choose the group composition was also praised by students in another evaluation of all courses in the winter semester 2021/22.

In summary, social skills such as collaboration and teamwork are developed when the task of producing an educational podcast is performed in a group. This confirms previous findings [6]. It is worth noting that social skills are unlikely to be promoted when podcasts are used as a substitute or supplement, highlighting the potential for their creative use. But there are many other skills that are fostered by using podcasts as information communication technology.

Students agreed in the questionnaire that their communication skills, their competence in using the technology itself, and their creativity are improved. This is consistent with previous reports on the impact of podcasts in higher education [4]. Interestingly, additional skills are enhanced through a collaborative learning process [5], indicating the power of podcasts as an educational tool. It was generally found that the creation of a podcast deepens the perception of the instructional content [4,14]. In the current study, respondents indicated that developing an educast was helpful to reflect on the learning content, but a neutral stance was inferred regarding the potential to actually support learning of the content. This explains why students are rather cautious about using educasts to prepare for the final exam. Neutral attitudes toward listening to podcasts on the way to university have been reported previously [16].

Podcasts seem useful for gaining a general overview of the subject or for revision [12], but rather less so for deep learning. The question has been raised before about whether podcasts are more effective for summaries than for extensive lectures [25], which often provide background and context. Students’ individual feedback also made clear that they felt podcasts had limited use for complex subject matter. Nonetheless, students appreciate creating an educast as a varied method of assessing performance. They had fun, enjoyed producing the educast, and their motivation was increased compared to the traditional writing of a protocol. Therefore, the students judged the developing of an educational podcast to be a stimulating learning task.

In the current case, this is probably due to the fact that the students were given almost no guidelines on how to implement the educast. In fact, there is literature that provides tips for creating a good podcast for educational purposes [22]. Others suggest that podcast creators should refer to theoretical frameworks to make the podcast as good as possible [7]. Nevertheless, it does not seem useful to instruct pharmacy students on how to create an effective podcast. What matters is the delivery of the course content. The podcast is only a means to an end. Here the podcast should only serve as an alternative to conventional and less creative methods of learning.

The introduction of podcasts in courses proved to be a positive experience, as previously reported [5]. This is perhaps due to the fact that in the course of their pharmacy studies so far, the respondents had almost no experience in the application of podcasts. In contrast, they even expressed the wish that more new techniques should be used during their studies. In the questionnaire, the students agreed to keep podcasts as a tool and to use them more often in other courses. They are not of the opinion that the podcasts should only be provided by the lecturers, but that students can also create them.

This very positive attitude describes the acceptance of podcasts by students. The overall effectiveness of podcasts in higher education depends critically on student perceptions [4,6]. This has proven successful in the present pilot study and, thus, principally allows the integration into pharmacy labs [10], unless the content is too comprehensive. Nonetheless, a comparative study should be conducted in the future to determine if students who created an educast instead of writing a protocol actually learned the subject better than students who did not make a podcast but prepared the usual protocol. Following research to continue this pilot study could investigate the dissemination of educasts to other learners and peers [6], as podcasts are valuable tools to disseminate knowledge [15].

This can remove some pressure from the instructor and induce self-directed teaching and learning among students. Aside from fostering competencies about content expertise among students by offering this tool, the docent also enjoys the benefit of podcasts being a diverse medium. Listening to the podcasts leads to variety with the docent because of the creative implementation by the students, rather than the monotonous correcting of protocols. Consequently it can lead to increased motivation on the part of the lecturer.

## 5. Conclusions

In recent years, the impact of using podcasts in higher education has increased. The demand for mobile learning methods during the current pandemic has highlighted the importance of this tool, as it allows for knowledge transfer in a safe and socially distant environment. Educational podcasts are primarily used to substitute or supplement lectures. In these cases, students listen to podcasts only passively. However, actively creating podcasts has an even more stimulating effect for students. This generated the idea to exploit educasts as a tool for performance assessment. Therefore, the current pilot study investigated the general extent to which podcasts are accepted by pharmacy students. Overall, there was a broad perception of this medium, which can be attributed on the one hand to the apparent underrepresentation of new technologies in pharmacy teaching. The students enjoyed creating the podcast. The results from the voluntary questionnaire indicated the strengthening of competencies, e.g., social skills. However, the students saw a limitation in the fact that the medium might not be optimally suited for complex topics, such as complicated pharmacology topics. Nevertheless, students are in favor of keeping it as a medium for performance assessment and they even suggest integrating it into other courses. It demonstrates the powerfulness of creating educasts by students in pharmacy education and that such media can also be used beneficially beyond mobile learning. This is another advantage gained from the redesign of teaching as a result of the COVID-19 pandemic and should be used in the future.

## Figures and Tables

**Table 1:** Respondent (n = 27) feedback to the questionnaire on the use of educational podcasts (educasts) as a medium in pharmacy studies.

Question (Q)	Score 1: Strongly Agree n (%)	Score 2: Agree n (%)	Score 3: Neutral n (%)	Score 4: Disagree n (%)	Score 5: Strongly Disagree n (%)	Mean Score
Q1: During my course of pharmacy studies so far, educasts have been used very intensively.	0 (0)	1 (3.7)	0 (0)	2 (7.4)	24 (88.9)	4.81
Q2: Principally, I think that much more new media should be used in university teaching.	6 (22.2)	12 (44.4)	9 (33.3)	0 (0)	0 (0)	2.11
Q3: Basically, I like audio podcasts.	4 (14.8)	13 (48.1)	7 (25.9)	2 (7.4)	1 (3.7)	2.37
Q4: Audiovisual podcasts are significantly better than merely audio podcasts.	5 (18.5)	8 (29.6)	10 (37.0)	2 (7.4)	2 (7.4)	2.56
Q5: In principle, I consider educasts to be a suitable tool for the professional processing of teaching content.	4 (14.8)	12 (44.4)	7 (25.9)	4 (14.8)	0 (0)	2.41
Q6: Production of an educast for performance assessment is a more varied method than writing conventional protocols. ^1^	14 (53.8)	8 (30.8)	2 (7.7)	2 (7.7)	0 (0)	1.69
Q7: Producing the educast allows me to focus on the essential learning content.	6 (22.2)	5 (18.5)	10 (37.0)	5 (18.5)	1 (3.7)	2.63
Q8: The production of the educast is helpful to reflect and elaborate on the learning content.	7 (25.9)	12 (44.4)	5 (18.5)	3 (11.1)	0 (0)	2.15
Q9: The production of the educast is helpful to learn the learning content.	3 (11.1)	11 (40.7)	3 (11.1)	6 (22.2)	4 (14.8)	2.89
Q10: When producing an educast, my creativity is encouraged.	14 (51.9)	11 (40.7)	0 (0)	1 (3.7)	1 (3.7)	1.67
Q11: For producing an educast, I prefer partner/group work.	16 (59.3)	3 (11.1)	7 (25.9)	1 (3.7)	0 (0)	1.74
Q12: For producing an educast, I prefer individual work.	0 (0)	3 (11.1)	6 (22.2)	7 (25.9)	11 (40.7)	3.96
Q13: Individual aspects of the students can be taken into account during producing educasts in a small group.	7 (25.9)	16 (59.3)	3 (11.1)	1 (3.7)	0 (0)	1.93
Q14: By producing an educast, students’ social skills can be promoted.	7 (25.9)	13 (48.1)	6 (22.2)	1 (3.7)	0 (0)	2.04
Q15: By producing an educast, students’ communication skills can be promoted.	7 (25.9)	16 (59.3)	3 (11.1)	1 (33.7)	0 (0)	1.93
Q16: By producing an educast, students’ media literacy can be promoted.	7 (25.9)	18 (66.7)	2 (7.4)	0 (0)	0 (0)	1.81
Q17: By producing an educast in a small group, students’ self-organization can be promoted.	8 (29.6)	13 (48.1)	5 (18.5)	1 (3.7)	0 (0)	1.96
Q18: The time required to produce an educast is too extensive.	1 (3.7)	1 (3.7)	6 (22.2)	12 (44.4)	7 (25.9)	3.85
Q19: In terms of necessary skills as a future pharmacist, the production of educasts represents a good exercise.	3 (11.1)	11 (40.7)	5 (18.5)	8 (29.6)	0 (0)	2.67
Q20: Educasts should preferably be produced by faculty and then provided to students, not the other way around.	0 (0)	6 (22.2)	4 (14.8)	11 (40.7)	6 (22.2)	3.63
Q21: The production of educasts can be kept as a tool for performance assessment.	6 (22.2)	11 (40.7)	5 (18.5)	5 (18.5)	0 (0)	2.33
Q22: I can imagine that the production of educasts could be integrated into other courses of my previous pharmacy studies.	6 (22.2)	11 (40.7)	7 (25.9)	3 (11.1)	0 (0)	2.26
Q23: I can imagine listening to educasts in order to prepare for the final exam.	3 (11.1)	10 (37.0)	4 (14.8)	7 (25.9)	3 (11.1)	2.89
Q24: I can imagine listening to educasts in my spare time...						
(a) …on the way to the university, when commuting to the university.	4 (14.8)	9 (33.3)	3 (11.1)	7 (25.9)	4 (14.8)	2.93
(b) …when jogging or during other sports activities.	0 (0)	5 (18.5)	4 (14.8)	8 (29.6)	10 (37.0)	3.85
(c) ...during household chores (cleaning, laundry, ironing, cooking).	9 (33.3)	12 (44.4)	1 (3.7)	4 (14.8)	1 (3.7)	2.11
(d) ...before falling asleep.	4 (14.8)	2 (7.4)	3 (11.1)	6 (22.2)	12 (44.4)	3.74
Q25: The duration of the educast of 3–5 min is too short.	2 (7.4)	2 (7.4)	10 (37.0)	9 (33.3)	4 (14.8)	3.41
Q26: The duration of the educast of 3–5 min is too long.	0 (0)	0 (0)	4 (14.8)	11 (40.7)	12 (44.4)	4.30
Q27: The topic (urine test strips/pulse oximetry) is suitable for the production of an educast.	16 (59.3)	11 (40.7)	0 (0)	0 (0)	0 (0)	1.41

^1^ Of all the returned questionnaires, one student did not answer this question (n = 26).

**Table 2:** Individual comments of the respondents.

Comment (C)	Text
C1	“Regarding when I would listen to the educast, only at home during the learning phase, if I listen to a 5 min educast before falling asleep I will understand little of it. Concerning duration I chose neutral, because that is very topic specific.”
C2	“Perhaps a bit too elaborate for a simple protocol submission, prefer to bring in other areas (e.g., instead of a lecture, etc.)”
C3	“In principle, the format of the educast is not a bad idea, as these can be listened to quite well on the side. Nevertheless, I personally find written reports better, because here you can search more specifically for keywords or the like and especially more complicated learning content is often better structured than you could summarize it purely verbally.”
C4	“For listening on the side, educasts could be quite useful, you definitely get a rough overview of the topic, general contexts can be remembered well. For pure factual knowledge (location of absorption maxima or reference ranges) I would not use educasts.”
C5	“I found the task, as free as it was set, very good, but I think that a more extensive task would make the creation of an educast more difficult and a protocol would then basically be easier again.”
C6	“For these two topics, a podcast was appropriate. For more complex topics, I always find a visual representation better.”
C7	“Should be used more often, directs the concentration on the content.”
C8	“Very varied method, very much fun. Please keep it.”
C9	“The fun factor in creating educast is definitely much higher than in protocol writing. This motivates you very much to take another look at the learning material. In my opinion, a very good project! :)”

## Data Availability

Not applicable.
